# Observation on the clinical effect of high-dose Intravenous Immunoglobulin combined with low-dose prednisone acetate in the treatment of patients with Kawasaki Disease

**DOI:** 10.12669/pjms.37.4.4023

**Published:** 2021

**Authors:** Hao Zhang, Mei-ying Wang, Yong-nan Teng, Xiao-dan Wang, Hai-tao Cao

**Affiliations:** 1 Hao Zhang, Department of Cardiology, Baoding City Children Respiratory and Digestive Diseases Clinical Research Key Laboratory, Baoding 071000, China. Baoding children’s Hospital, Baoding 071000, China; 2 Mei-ying Wang, Department of Laboratory Medicine, Baoding children’s Hospital, Baoding 071000, China; 3 Yong-nan Teng, Department of Gastroenterology, Baoding children’s Hospital, Baoding 071000, China; 4 Xiao-dan Wang, Department of Anesthesiology, Baoding children’s Hospital, Baoding 071000, China; 5 Hai-tao Cao, Department of Laboratory Medicine, Army 82nd Group Military Hospital, Baoding 071000, China

**Keywords:** HDIVIG, Low-dose methylprednisone, Kawasaki disease (KD), Pediatrics, Treatment

## Abstract

**Objective:**

To evaluate the clinical effect of high-dose intravenous immunoglobulin (HDIVIG) single dose and pulse therapy combined with small-dose prednisone acetate in the treatment of patients with Kawasaki disease (KD).

**Methods:**

Eighty patients with KD from Baoding Children’s Hospital, China, were randomly divided into two groups: the experimental group and the control group, each with 40 cases. Patients in the experimental group were treated with HDIVIG single dose, pulse therapy combined with low-dose prednisone acetate, while patients in the control group were treated with conventional-dose immunoglobulin. Patients in both groups were treated with aspirin orally, and given symptomatic treatment including anti-inflammatory, nutritional support, correction of water and electrolyte disturbance and acid-base balance. Peripheral venous blood samples were drawn from all patients at the time of admission, Day-1, Day-7 and Day-14 after treatment, and in the basic state of getting up in the morning, and then the levels of tumor necrosis factor (TNF-a), C-reactive protein (CRP), interleukin-6 (IL-6) and other inflammatory factors were detected by enzyme-linked immunosorbent assay (ELISA). The time of body temperature falling to normal, lymph node swelling recovery, hands and feet swelling, mucosal hyperemia regression after treatment in the two groups was recorded, and the treatment effect of the two groups was comprehensively evaluated.

**Results:**

After treatment, the levels of inflammatory factors such as TNF-a, CRP, IL-6 in the experimental group were significantly lower than those in the control group, with a statistically significant difference (P<0.05). In addition, the time of body temperature falling to normal, lymph node swelling recovery, hands and feet swelling, and mucosal hyperemia regression in the experimental group was significantly shorter than that in the control group (p=0.00). The effective rate of the experimental group was 95% and that of the control group was 80%, with a statistically significant difference (p=0.04).

**Conclusion:**

HDIVIG single dose, pulse therapy combined with small-dose prednisone acetate has a favourable therapeutic effect in the treatment of patients with KD, by which the inflammatory factors can be significantly improved, clinical symptoms and weight can be quickly ameliorated, and therapeutic effect can be enhanced.

## INTRODUCTION

Kawasaki disease (KD) is an acute febrile systemic vasculitis mainly occurring in children under five years old.^1^ Its etiology is uncertain, and there may be a complex interaction of genetic, infection and immune factors.^2^ This disease is clinically manifested as skin, mucous membrane and lymph node involvement in multiple parts of the body as well as high fever, which may lead to coronary artery damage or even life-threatening if not treated in time.^3^ Despite the fact that intravenous immunoglobulin is the main means of initial treatment and is widely applied clinically, there are still about 20% of patients whose curative effect is not very accurate and need to be treated with other treatment regimens.^4^ Aspirin has gained attention for its anti-inflammatory and antipyretic effects. Corticosteroids combined with IVIG intravenous application and aspirin have been proven in the literature to have better therapeutic advantages for patients.^5^ In this experiment, HDIVIG single dose, pulse therapy combined with low-dose prednisone acetate oral administration was used to treat patients with KD, and obvious clinical effects were achieved.

## METHODS

### Ethical approval

The study was approved by the Institutional Ethics Committee of Baoding children’s Hospital at December 15^th^, 2020 and written informed consent was obtained from all participants.

### Inclusion criteria:

1. All patients who meet the diagnostic criteria of KD.^6^
2. Patients under 14 years old.
3. Patients without other treatment before admission.
4. Patients whose family members are willing and able to cooperate to complete the study.
5. Patients who sign informed consent forms.

### Exclusion criteria:

1. Patients with severe organic or congenital diseases of heart, liver and kidney.
2. Patients allergic to immunoglobulin and prednisone.
3. Patients who withdraw from the study due to intolerable side effects of drugs.
4. patients with mental system diseases who cannot cooperate to complete the study.
5. Patients who take related drugs affecting the study such as immunosuppressants in the near future.

Eighty patients with KD admitted to our hospital were randomly divided into two groups with 40 cases in each group. Twenty-two males and 18 females were assigned to the experimental group, aged 1.5 -7.3 years old, with an average age of 4.68±2.40 years old, while 20 males and 20 females were assigned to the control group, aged 2.3 - 7.0 years old, with an average age of 4.53±2.32 years old. There was no significant difference in general data between the two groups, which were comparable (Table-I).

**Table-I:** Comparative analysis of general data between experimental group and control group (*X̅* S) n=40

Indicators	Experimental group	Control group	t/χ^2^	P
Age	4.68±2.40	4.53±2.32	0.15	0.88
Male (%)	22(62.5%)	20(57.5%)	0.20	0.65
Weight (kg)	26.53±5.40	25.70±3.99	0.78	0.44
Fever duration (d)	3.46±1.20	3.75±1.07	1.14	0.26

P>0.05

### Treatment methods

Patients in both groups were given symptomatic treatment such as anti-inflammatory, nutritional support, correction of water electrolyte disorder and acid-base balance. At the same time, aspirin was taken orally at a dose of 40 mg/kg three times a day, which gradually decreased to 3-5 mg/kg·d after the fever subsided for three days, and maintained for 6-8 weeks or until the indicators such as blood routine and erythrocyte sedimentation rate returned to normal.^7^ The experimental group was given HDIVIG combined with low-dose prednisone acetate on the basis of the above treatment. The specific treatment regimen was as follows: immunoglobulin 2g/kg, intravenous drip, single dose application. At the same time, prednisone acetate tablets were taken orally at 2mg/kg/d. The dosage was decreased according to the patient’s treatment, and the total course of hormone treatment is two weeks. In contrast, the control group received an intravenous drip of immunoglobulin 1g/kg/d for 2d (inconsistent with the guidelines, where the dose was given at 2g/kg/d).

### Observation indicators:

### Inflammation indicators

Peripheral venous blood samples were drawn from all patients at the time of admission, Day-1, Day-7 and Day-14 after treatment, and in the basic state of getting up in the morning, and then the levels of tumor necrosis factor (TNF-a), C-reactive protein (CRP), interleukin-6 (IL-6) and other inflammatory factors were detected by enzyme-linked immunosorbent assay (ELISA).

### Time for clinical symptoms and signs to disappear

The time of body temperature falling to normal, cervical lymph node swelling recovery, hands and feet swelling, and conjunctival and oral mucosal congestion regression after treatment in the two groups were recorded, and the differences in the improvement time of symptoms and signs between the two groups were compared and analyzed.

### Efficacy evaluation

according to the guideline of diagnosis and treatment of Kawasaki disease compiled by Ishii et al., clinical symptoms and laboratory examination were combined to evaluate the two groups of patients. Judgment criteria: markedly effective: symptoms disappeared within 5d after treatment, and various laboratory indicators return to normal.

### Efficacy

symptoms disappeared and laboratory indicators improved significantly after 5-8 days of treatment. The symptoms were still obvious or even worse, and laboratory indicators showed no improvement after twelve days of treatment. Total effective rate = (markedly effective + effective)/total number of cases x 100%.^8^

### Statistical analysis

All the data were statistically analyzed by SPSS 20.0 software, and the measurement data were expressed as (*X*¯± s). Two independent sample t-test was used for inter-group data analysis, repeated measurement analysis of variance was used for intra-group data analysis, and 2-test was adopted for rate comparison. P<0.05 indicates a statistically significant difference.

## RESULTS

The changes in inflammatory factors before and after treatment between the two groups are shown in Table-II, indicating that TNF-a, CRP, IL-6 and other inflammatory factors in the two groups were significantly increased before treatment, and the difference was not significant (p>0.05). The above indicators decreased after treatment, with a statistically significant difference (p<0.05). The levels of TNF-a, CRP and IL-6 in the experimental group were significantly lower than those in the control group (Day-I after TNF treatment, p=0.02, on Day-7 and Day-14, p=0.00; Day-1, Day-7 and Day-14 after CRP and IL-6 treatment, p=0.00).

**Table-II:** Comparative analysis of changes in inflammatory factors before and after treatment between the two groups (*X̅*±S) n=40.

Group		Before treatment*	1d after treatment	7d after treatment Δ	14d after treatment Δ	F	P
TNF-ɑ(ng/L)	Experimental group Δ	43.23±10.25	22.30±9.41	6.13±1.54	4.82±2.01	23.22	0.00
	Control group Δ	44.03±10.57	27.54±9.57	10.35±4.37	7.11±3.02	21.24	0.00
	t	0.34	2.47	5.76	3.99		
	p	0.73	0.02	0.00	0.00		
CRP(mg/L)	Experimental groupΔ	84.02±17.01	14.72±5.36	6.73±1.32	4.01±0.76	29.72	0.00
	Control group Δ	83.73±16.72	22.03±6.49	10.25±5.17	5.17±1.20	29.64	0.00
	t	0.08	5.49	4.17	5.16		
	p	0.93	0.00	0.00	0.00		
IL-6(ng/L)	Experimental group Δ	15.65±5.43	9.23±1.52	6.47±2.27	2.36±0.44	15.43	0.00
	Control group Δ	16.25±4.85	12.55±3.42	9.82±2.35	5.32±1.02	13.95	0.00
	t	0.52	5.61	6.48	42.47		
	p	0.60	0.00	0.00	0.00		

* p>0.05, Δp<0.05

The time of body temperature falling to normal, cervical lymph node swelling recovery, hands and feet swelling, and conjunctival and oral mucosal congestion regression after treatment in the experimental group were significantly shorter than those in the control group, indicating that the above indicators in the experimental group recovered faster, with a statistically significant difference (p=0.00) (Table-III).

**Table-III:** Comparative analysis of clinical symptoms recovery time (d) between the two groups after treatment (*X̅* ±S) n=40

Group	Conjunctiva and oral mucosa congestion	Hands and feet swelling	Cervical lymph node swelling	Body temperature recovery
Experimental group	3.32±1.48	4.43±1.72	3.51±1.46	1.47±0.32
Control group	5.74±2.75	6.03±1.05	4.79±1.77	3.22±1.08
t	4.90	5.02	3.53	9.83
p	0.00	0.00	0.00	0.00

p<0.05

The number of markedly effective, effective and ineffective cases of the two groups were recorded respectively within fourteen days after treatment according to the guidelines for diagnosis and treatment of Kawasaki disease compiled by Ishii et al. The effective rate of the experimental group was 95%, which was obviously superior to 80% of the control group (p=0.04) (Table-IV).

**Table-IV:** Comparative analysis of therapeutic efficiency rate between the two groups (*X̅* ±S) n=40

Group	Markedly effective	Effective	Ineffective	Effective rate
Experimental group	32	6	2	95%
Control group	25	7	8	80%
χ^2^				4.11
p				0.04

p<0.05

## DISCUSSION

Kawasaki disease (KD) is a common vasculitis disease usually seen in children below five years old. Pathogen infection is a potential inducement for susceptible children.^9^ It is believed by Marrani et al. that patients with KD have a strong innate immune response, which causes inflammatory damage to systemic blood vessels.^10^ However, unlike most infectious diseases, KD differs considerably among different races, indicating that genetics has a certain pathogenic mechanism in the disease.^11^ It has been identified by Onouchi via genome-wide association and genome-wide linkage studies that several susceptibility genes (such as ITPKC, CASP3, CD40 and ORAI) and chromosomal regions have a bearing on KD.^12^ Genetic testing is conducive to facilitating the early diagnosis and treatment of KD, and reducing improper treatment of patients diagnosed with other diseases.^13^ Coronary artery involvement, as the paramount complication of KD, may lead to severe coronary artery stenosis and further cause ischemic heart disease.^14^

Clinically, intravenous immunoglobulin combined with aspirin is regarded as the principal therapeutic regimen of KD.^15^ With the combination of intravenous immunoglobulin (IVIG) and aspirin, the incidence of coronary artery disease in sick children was greatly reduced.^16^ Specifically, aspirin is a non-steroidal anti-inflammatory drug. Its effects and advantages include: antipyretic and analgesic effects; inhibition of prostaglandin-mediated inflammation and vasculitis; aggregation of platelets and control of coronary artery injury to a certain extent. Moreover, aspirin can play its role not only in regulating the hypothalamic thermoregulation center, but also in effectively controlling patient’s body temperature, showing relatively high safety for patients with KD.^17^ Synergistic effects can be achieved by the combined application of immunoglobulin and aspirin in alleviating symptoms and improving patients’ inflammatory response. However, no uniform standard has been established regarding the dosage and period of administration of immunoglobulin. It is claimed by Dietz et al.^18^ that the application dose of immunoglobulin should be a high dose to quickly control the condition and relieve symptoms. According to the research of Dionne et al.^19^, high-dose IVIG (2g/kg, single application) and conventional-dose (1g/kg) IVIG can not only exert a more advantageous clinical therapeutic effect, but also be safe and effective. At the same time, such a treatment regimen also has a very favorable preventive effect on coronary artery disease, and can reduce its incidence to less than 5%.^20^

The mechanism of vascular injury in patients with KD is believed to be related to the activation of monocyte/macrophage system in vivo, which releases a large number of inflammatory mediators and inflammatory factors and causes vascular endothelial injury.^21^ It has been shown in research^22^ that TNF-a can stimulate T cells to produce various inflammatory factors and aggravate the inflammatory response, which plays an important part in the inflammatory response and immune regulation of the body. CRP increased dramatically in the early stage of inflammatory reaction. IL-6 is a proinflammatory factor that can stimulate the proliferation and differentiation of T cells and B cells, increase the release of inflammatory factors and aggravate the inflammatory reaction. It was further confirmed in this research that the serum levels of the above inflammatory factors were significantly increased in patients with KD before treatment, indicating the presence of a high level of inflammatory response in the body. Consequently, reducing the level of inflammatory factors and protecting vascular endothelial function are also key to the treatment of KD.

Despite the controversy over the role of corticosteroids^23^, more literature supports that certain benefits may be obtained by glucocorticoids in the treatment of the disease.^24^ According to research conducted by Gamez et al., the first-line treatment of KD includes intravenous immunoglobulin and corticosteroids, and that low-dose, short-term hormone therapy has lower side effects and is easier to be accepted.^25^ It has further been confirmed by the research of Miyat et al. that the side effects of low-dose prednisone acetate are relatively small.^26^

It is confirmed by our research that on the basis of oral administration of aspirin, the reduction of inflammatory factors such as TNF-a, CRP and IL-6 in patients treated with HDIVIG high dose, pulse therapy combined with low-dose prednisone acetate was more significant than that of conventional immunoglobulin therapy (p<0.05), suggesting that the treatment regimen combined with corticosteroids is more conducive to reducing the inflammatory state in patients. Teraura believes that serum IL can be used as an effective marker of disease activity and therapeutic effect in patients with KD, and its reduction is of great significance for the efficacy and prognosis of patients.^27^ Moreover, the time of body temperature falling to normal, lymph node swelling recovery, hands and feet swelling, and mucosal hyperemia regression in the experimental group was significantly shorter than that in the control group (p=0.00), suggesting that the inflammatory reaction disappeared faster than the control group, which indirectly confirmed the benefits of reducing the high inflammatory state in the body for patients. Therefore, the therapeutic efficiency rate of the experimental group was significantly higher than that of the control group (95% vs. 80%), with a statistically significant difference (p=0.04).

### Limitations of the study

Nevertheless, deficiencies still exist in this research: the sample size is relatively small, only the indicators of patients after treatment is compared and analyzed, and there are no strict long-term follow-up results. In addition, no separate comparative analysis has been conducted on patients with coronary artery injury due to the small sample size. In our future research work, corresponding measures will be taken to further expand the sample size, increase the follow-up time after treatment, and further improve the treatment data of patients with coronary artery damage, so as to elaborate the long-term effect of the treatment scheme and the prognosis of patients with heart damage.

## CONCLUSION

HDIVIG single dose, pulse therapy combined with small-dose prednisone acetate has a good therapeutic effect in the treatment of patients with KD, by which the inflammatory factors can be significantly improved, clinical symptoms and weight can be quickly ameliorated, and therapeutic effect can be enhanced.

### Authors’ Contributions:

**HZ and** **MYW** designed this study and prepared this manuscript,and are responsible and accountable for the accuracy or integrity of the work.

**YNT** **and** **HTC** collected and analyzed clinical data.

**XDW** significantly revised this manuscript.
